# Decipher the ancestry of the plant-specific *LBD* gene family

**DOI:** 10.1186/s12864-016-3264-3

**Published:** 2017-01-25

**Authors:** Yimeng Kong, Peng Xu, Xinyun Jing, Longxian Chen, Laigeng Li, Xuan Li

**Affiliations:** 1 Key Laboratory of Synthetic Biology, CAS Center for Excellence in Molecular Plant Sciences, Institute of Plant Physiology and Ecology, Shanghai Institutes for Biological Sciences, Chinese Academy of Sciences, Shanghai, 200032 China; 2 National Key Laboratory of Plant Molecular Genetics, CAS Center for Excellence in Molecular Plant Sciences, Institute of Plant Physiology and Ecology, Shanghai Institutes for Biological Sciences, Chinese Academy of Sciences, Shanghai, 200032 China

**Keywords:** *LBD* gene family, Phylogenetic analysis, Land plant, Evolution

## Abstract

**Background:**

*Lateral Organ Boundaries Domain* (*LBD*) genes arise from charophyte algae and evolve essential functions in land plants in regulating organ development and secondary metabolism. Although diverse plant species have been investigated to construct the phylogeny of *LBD* gene family, a detailed and reliable ancestry that characterizes their evolutionary patterns has not been revealed.

**Results:**

We develop an improved bioinformatic method that allows robust detection of 431 *LBD* genes in 11 high-quality land plant genomes. Phylogenetic analysis classifies the *LBD* genes into six subfamilies which support the existence of 7 ancient gene lineages. Phylogenetic relationship and gene collinearity are combined to retrace 11 ancestor genes for seed plants and 18 ancestor genes for angiosperms, which improves the resolution of *LBD* gene ancestry. The ancient gene lineages are strictly preserved in current plant genomes, including the previously controversial class IB gene in *Selaginella moellendorphii*, suggesting extreme reluctance of *LBD* genes to be lost during evolution. Meanwhile, whole-genome and dispersed gene duplications substantially expand *LBD* gene family in angiosperms, and elaborate functions of *LBD* genes through frequent expression pattern change and protein sequence variation.

**Conclusions:**

Through phylogenetic and gene collinearity analyses, we retrace the landscape of *LBD* gene ancestry which lays foundation for elucidating evolutionary diversification of *LBD* genes in land plants.

**Electronic supplementary material:**

The online version of this article (doi:10.1186/s12864-016-3264-3) contains supplementary material, which is available to authorized users.

## Background

Lateral Organ Boundaries (LOB) Domain (LBD) proteins define a group of plant-specific transcription factors that originated from charophyte algae [1, 2]. They share a characteristic LOB domain (also referred to as AS2 domain) with a conserved C-motif (CX2CX6CX3C), a Gly–Ala–Ser (GAS) block, and a leucine-zipper-like coiled-coil motif [3–5]. The C-motif is predicted to bind to DNA sequence, while the coiled-coil motif functions in mediating protein-protein interaction. In *Arabidopsis*, *LBD* genes were first identified for the specific expression at the base of lateral organs and the noticeable function in regulating leaf development [3, 4]. Subsequent studies showed diverse functions of *LBD* genes in regulating plant organ development and secondary metabolism: *AtLBD6/AtAS2* regulates leaf formation [3]; *AtLBD16*, *AtLBD18* and *AtLBD29* control lateral root formation [6]; *AtLBD27/SCP* is required for microspore cell divisions [7]; *AtLBD37*, *AtLBD38* and *AtLBD39* are negative regulators of anthocyanin biosynthesis and N availability signals [8]. It is frequently observed that *LBD* genes exhibit similar biological functions in different angiosperm species. For example, *AtLBD16* orthologs control lateral root formation in *Arabidopsis*, rice and maize [6, 9, 10], and *AtLBD6/AtAS2* orthologs repress meristematic cell formation and regulate abaxial-adaxial leaf polarity in *Arabidopsis* and maize [11–14]. The conserved roles of *LBD* gene orthologs suggest their functions may have been established prior to angiosperms emergence.

Extensive efforts have been exerted to classify *LBD* gene family in diverse plant species. In *Arabidopsis*, two major classes of *LBD* genes are traditionally classified according to the LOB domain structure [3, 4]. Class II proteins are distinctive to the class I due to the absence of the coiled-coiled motif. Further subdivisions of the two major classes reveal highly dynamic patterns in different species. In rice and maize, five subgroups are divided among class I *LBD* genes [15, 16]. In *Arabidopsis* and *Malus*, four and nine subgroups of class I *LBD* genes are classified respectively [5, 17]. The inconsistent subgroup number in these studies is likely caused by extensive duplications of *LBD* genes in angiosperms, and a single plant genome may not encompass their full diversity.

Recent studies performed more comprehensive analysis of phylogenetic distribution of *LBD* genes in multiple species spanning bryophytes, lycophytes and seed plants [1, 2]. Their results accordantly subdivide *LBD* genes into class IA, IB, IC1/ID, IC2, IE and class II, but there are still debates on whether class IB *LBD* genes are present in the genome of lycophytes [1, 2]. Moreover, angiosperm genomes retain large-scale collinear gene blocks which provides direct evidence to identify orthologous genes descendent from a common ancestor [18]. But none of above studies investigates collinearity of *LBD* genes to infer their ancestors at each stage of land plant evolution. Therefore, a detailed and reliable ancestry that describes evolutionary history of *LBD* genes has not been revealed. Here we develop an improved method for *LBD* gene detection in 11 representative plant species. Through investigating gene collinearity and phylogenetic relationships, we present a detailed ancestry of *LBD* genes which characterizes their diversifying patterns in land plants.

## Results and discussion

### An improved method to identify *LBD* genes

**Fig. 1:**
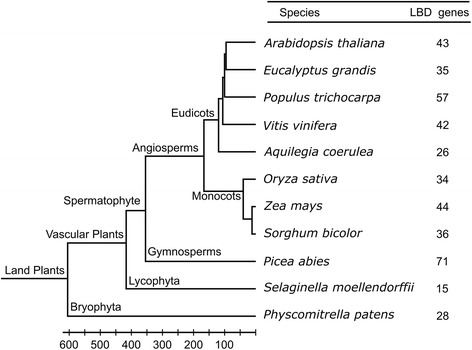
Summary of *LBD* genes in the analyzed land plant species. The order of tree branches and divergence time are derived from the TimeTree database (http://timetree.org/)

Previous strategy to detect *LBD* genes is primarily based on database query or BLAST search of *Arabidopsis* protein sequences [1, 2], which heavily depends on *Arabidopsis* sequence features and has limited applications for evolutionarily divergent genomes of land plants. Therefore we develop a new method that can be widely employed to detect *LBD* genes. As sequences of LBD proteins are highly variable except the conserved LOB domain [3, 4], we use the PFAM profile hidden Markov models of the LOB domain (PFAM database, http://pfam.xfam.org/) to query each plant genome with a cutoff of gathering threshold (E-value 1e-5). Only proteins with matched sequence covering at least 80% of the LOB domain are regarded as LBD proteins. This improved method would be effective to preclude partial sequence matches outside the LOB domain, while allowing identification of *LBD* genes with relative sequence variance. We identify 431 *LBD* genes in 11 high-quality genomes of land plants spanning bryophytes to angiosperms (Fig. 1 and Additional file 1). The lycophyte *Selaginella* contains 15 *LBD* genes, ranking the least in land plants. The number of *LBD* genes nearly doubles in bryophyte *Physcomitrella* which has a genome duplication event [19]. Apart from basal eudicot *Aquilegia*, more than 34 *LBD* genes are identified in the angiosperm genomes (Fig. 1), suggesting extensive expansion of *LBD* gene family in angiosperms. Noticeably, compared with the previous study [1], our improved method identifies more *LBD* genes in basal-node bryophyte *Physcomitrella* (28 vs 26) and lycophyte *Selaginella* (15 vs 11).

### Expansion patterns of *LBD* genes in angiosperms

**Fig. 2:**
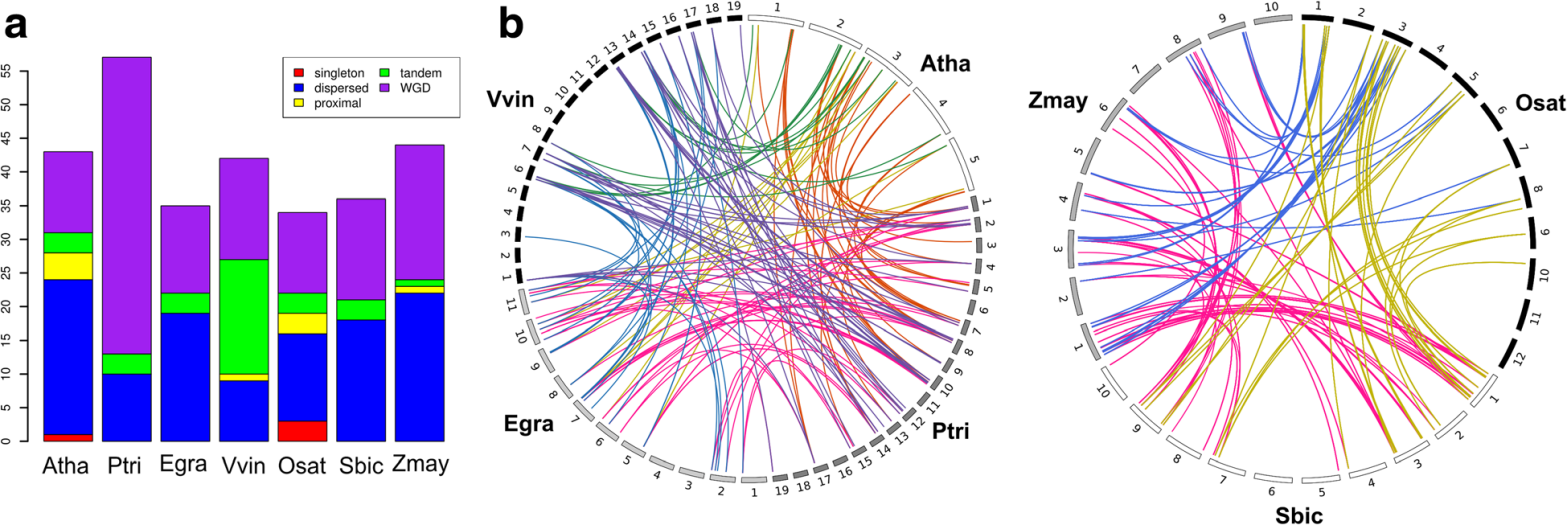
A category of *LBD* gene duplication events and inter-species collinearity. **a** The histogram indicates the occurrence of *LBD* gene duplication events in representative angiosperm species. **b** Inter-species collinearity of *LBD* genes in dicots and monocots. Colored lines represent pairs of orthologous *LBD* genes in the conserved syntenic blocks of plant genomes

The expansion of *LBD* genes in angiosperms suggests they are likely influenced by whole-genome duplications (WGDs) that substantially increase gene content [18]. Therefore, we analyze each angiosperm genome to identify different types of gene duplications that contribute to the diversity of *LBD* genes. According to genome positions of the affected genes, gene duplication events are categorized into different sorts: WGD, dispersed duplication, and tandem/proximal duplications. On average 84% of *LBD* genes in angiosperms are affected by WGD and dispersed duplications (Fig. 2a). WGD events influenced 35–45% of *LBD* genes in most angiosperm species, while the ratio can vary dynamically from 28% in *Arabidopsis* to 77% in *Populus*. This could be explained by the fact that *Arabidopsis* has lost many *LBD* gene duplicates following two recent WGDs within the crucifer lineage, whereas *Populus* retained more duplicated genes after the Salicaceae WGD event [20, 21]. Compared with WGD event, tandem duplications in *Populus* only affect 5.2% of *LBD* genes. In contrast, up to 40% of *LBD* genes in *Vitis* are influenced by tandem duplications, suggesting tandem duplication in *Vitis* is an important driving force for the expansion of *LBD* genes.

Previous studies demonstrate genes descendent from a common ancestor often share chromosomal collinearity in angiosperms [18]. Therefore, we analyze collinearity relationship of *LBD* genes to infer their inter-species orthology. Through iterative clustering of collinear *LBD* genes, we merge 133 *LBD* genes of dicots into 21 collinear groups and 92 *LBD* genes of monocots into 23 collinear groups (Fig. 2b and Additional file 2). The collinear groups contain 77% of total *LBD* genes in the analyzed angiosperms and each group represents a set of *LBD* gene orthologs originated from a common ancestor. Merging collinear groups into a higher hierarchy is not feasible because extensive genome fragmentation and rearrangement obscured syntenic blocks between dicots and monocots [18]. Although the result identifies less than 23 members of *LBD* gene ancestors, additional phylogenetic information is needed to collapse the collinear groups of dicots and monocots to reflect a common angiosperm ancestor.

### Retracing *LBD* gene ancestors in land plants

**Fig. 3:**
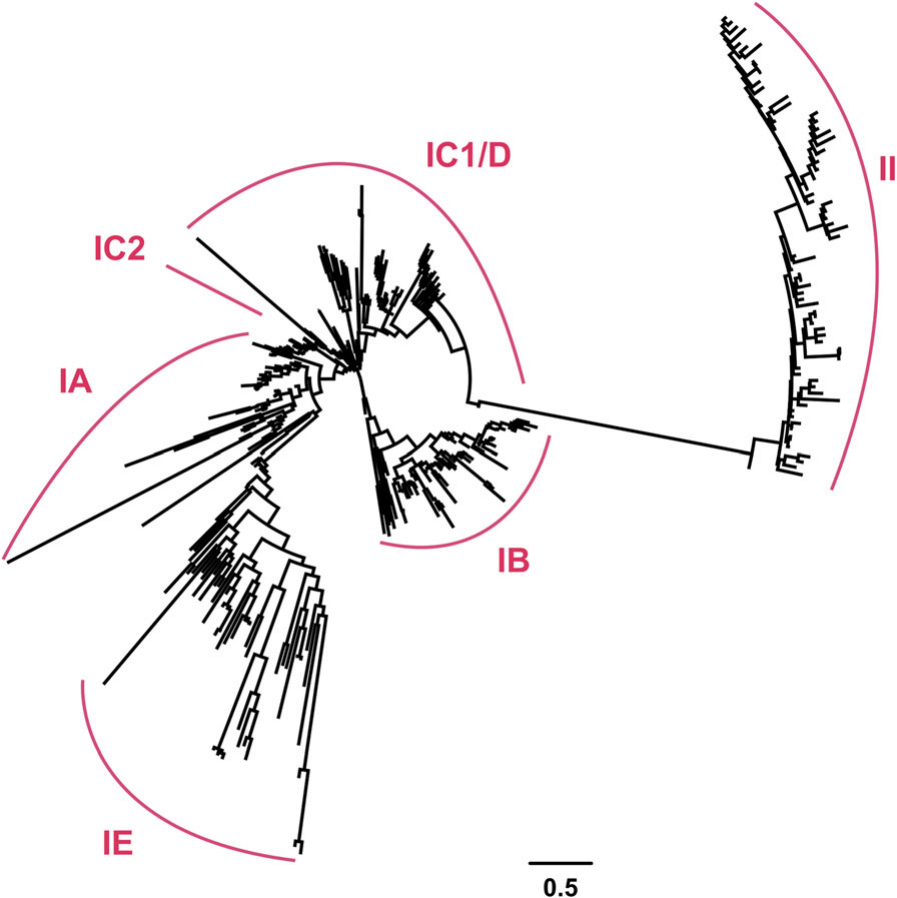
ML tree of *LBD* genes in land plants. The phylogenetic tree is constructed using the aligned LOB domains

A maximum likelihood (ML) tree is constructed with the aligned LOB domain in the 11 species to reveal phylogenetic relationships of *LBD* genes. The phylogenetic tree classifies *LBD* genes into class IA, IB, IC1/ID, IC2, IE, and class II, a topology similar with previously reported [1, 2] (Fig. 3). Through scrutinizing the phylogenetic tree, we identify 7 independent gene clusters of *Physcomitrella* and *Selaginella* genes that neighbor with *LBD* genes of all analyzed seed plants, suggesting they are ancient gene lineages that give rise to *LBD* genes in modern plant genomes. We detect two ancient lineages in class IA, and one ancient lineage in each of the five remaining classes (Additional file 3). Therefore, class IA is actually composed of two founder genes in early land plants. To yield a better resolution, we generate another ML tree using complete protein sequences of class IA. The phylogenetic tree verifies the existence of two ancient lineages with high support values (Shimodaira-Hasegawa-like approximate likelihood-ratio test (SH-aLRT) > 94%) (Additional file 4).

**Fig. 4:**
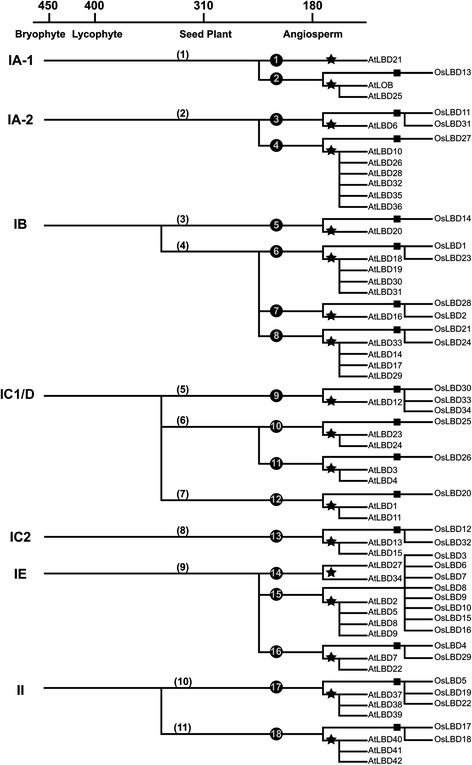
Reconstruction of *LBD* gene ancestry in land plants. The *LBD* genes in *Arabidopsis* and rice are mapped to the ancestral lineages at each key node of land plant evolution. Each line indicates a lineage of *LBD* genes. Asterisks and boxes and indicate the support from collinear gene groups in dicots and monocots. Note that the exact timing of duplication events is not estimated in the analysis due to uncertainties of the molecular clock assumption. The *LBD* genes in *Arabidopsis* are named in accordance with the previous study [4]

We then search gene clusters of seed plants to retrace ancestor *LBD* genes predating seed plants emergence. As phylogenetic tree often suffers from long branch attraction in which distant protein sequences are incorrectly grouped together [22], we employ stringent criteria in the analysis, requiring that *LBD* genes of gymnosperm *Picea* should be clustered with angiosperm genes from all the analyzed species. In total 11 gene clusters of seed plants were identified from the ML tree: three in class IC1/ID, two in class IA, IB and II, one in class IC2 and IE (Fig. 4 and Additional file 3). Because each cluster of seed plant *LBD* genes share the same phylogenetic origin, 11 *LBD* gene ancestors likely existed prior to seed plant divergence. Compared with the ancient lineages, class IB, IC1/ID and II *LBD* genes experience gene content expansion before seed plant evolution.

Gene collinearity and phylogenetic relationships are combined to retrace angiosperm ancestors of *LBD* genes. The ML tree categorizes 18 angiosperm gene clusters that include both dicot and monocot *LBD* genes (Fig. 4 and Additional file 3). Most of them are supported by inter-species gene collinearity except one cluster in class IE, which may be caused by massive gene duplications that likely obscure the syntenic block detection. Therefore, we propose that 18 *LBD* gene ancestors have been established prior to angiosperm emergence.

### Reconstruct the ancestry of *LBD* gene family

We reconstruct evolutionary history of *LBD* genes with the deduced ancestor genes. In early land plants, 7 ancient lineages of *LBD* genes were established and remained in a stable amount until the divergence of seed plants (Fig. 4). Two rounds of gene duplication occurred before the emergence of seed plants and angiosperms, expanding *LBD* gene family to 11 and 18 members respectively. Further expansion of *LBD* genes in individual angiosperm species is highly associated with WGDs. The basal eudicot *Aquilegia* contains 26 *LBD* genes, while the amount could be increased to over 40 members in other eudicots which have undergone genome triplication or additional WGD events (Fig. 1) [23]. The analyzed monocots have survived from two successive WGDs and contain more than 34 *LBD* genes in the genome [24]. Noticeably, all of the major lineages of *LBD* genes could be detected in current angiosperm genomes, indicating they are extremely reluctant to be lost during evolution.

**Fig. 5:**
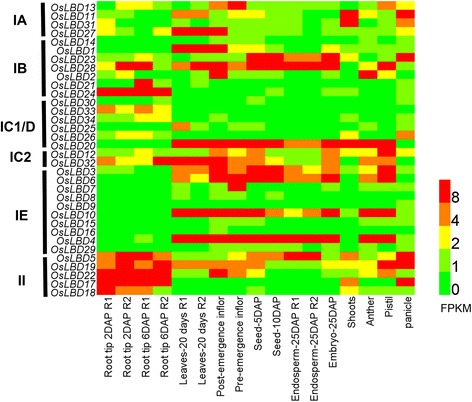
Tissue expression patterns of *LBD* genes in rice. The heatmap is constructed to illustrate expression level of 34 rice LBD genes. The RNA-seq data is obtained from the CARMO database [25]. The gene order is sorted according to their affiliated subfamilies

The reconstructed ancestry describes detailed evolutionary routes for 43 *LBD* genes in *Arabidopsis* and 34 *LBD* genes in rice. RNA-seq data of rice developmental atlas was further analyzed to investigate expression patterns of *LBD* genes from different subfamily [25]. The heatmap shows that different subfamily genes exhibit variable expression enrichment in different tissues (Fig. 5). For example, class IA *LBD* genes are more abundant in leaf and flower tissues, while class II *LBD* genes are universally expressed in diverse tissues. Meanwhile, it is often observed that *LBD* genes from a same subfamily genes display differential expression patterns. A noticeable case is in class IE which contains eight *LBD* genes (*OsLBD3*, *OsLBD6*, *OsLBD7*, *OsLBD8*, *OsLBD9*, *OsLBD10*, *OsLBD15* and *OsLBD16*) sharing a same angiosperm ancestor. Only four of these *LBD* genes show abundant expression in normal growth tissues, while the other genes are detected with very weak expression level (Fig. 5), suggesting expression pattern has been shifted during these genes evolution. Another case is the class IA-2 subfamily which contains *OsLBD11*, *OsLBD31* and *OsLBD27* paralog genes. Expression of *OsLBD11* is enriched in leaves, shoots and panicle (Fig. 5). Meanwhile, *OsLBD31* and *OsLBD27* display complementary expression patterns in either leaves or shoot and panicle, suggesting the two paralog genes underwent expression specialization after gene duplication. In *Arabidopsis*, similar expression divergence is also observed for class IA-2 *LBD* genes. For example, *AtLBD6/AS2* is specifically expressed in the adaxial side of leaves [3], while *AtLBD10* is only detected in the pollen grains, and *AtLBD36* is expressed in a variety of tissues, including leaf vasculature, flower organs and seeds [26, 27]. These observations suggest alteration of expression level and tissue specificity occurred during *LBD* genes evolution.

### Diversification of *LBD* gene subfamily

To deepen annotation and classification of *LBD* gene family, we developed sequence profile features for each *LBD* gene class using whole protein sequences alignment. The result shows that, besides the well recognized LOB domains, different gene classes possess specific characteristic protein sequences (Additional file 5). The location of these characteristic sequences varies among different classes. They can lie immediately downstream the LOB domain (class IA), present at C-terminal regions (class IB and IC1/ID), or extend flanking the LOB domain (class IC2).

**Fig. 6:**
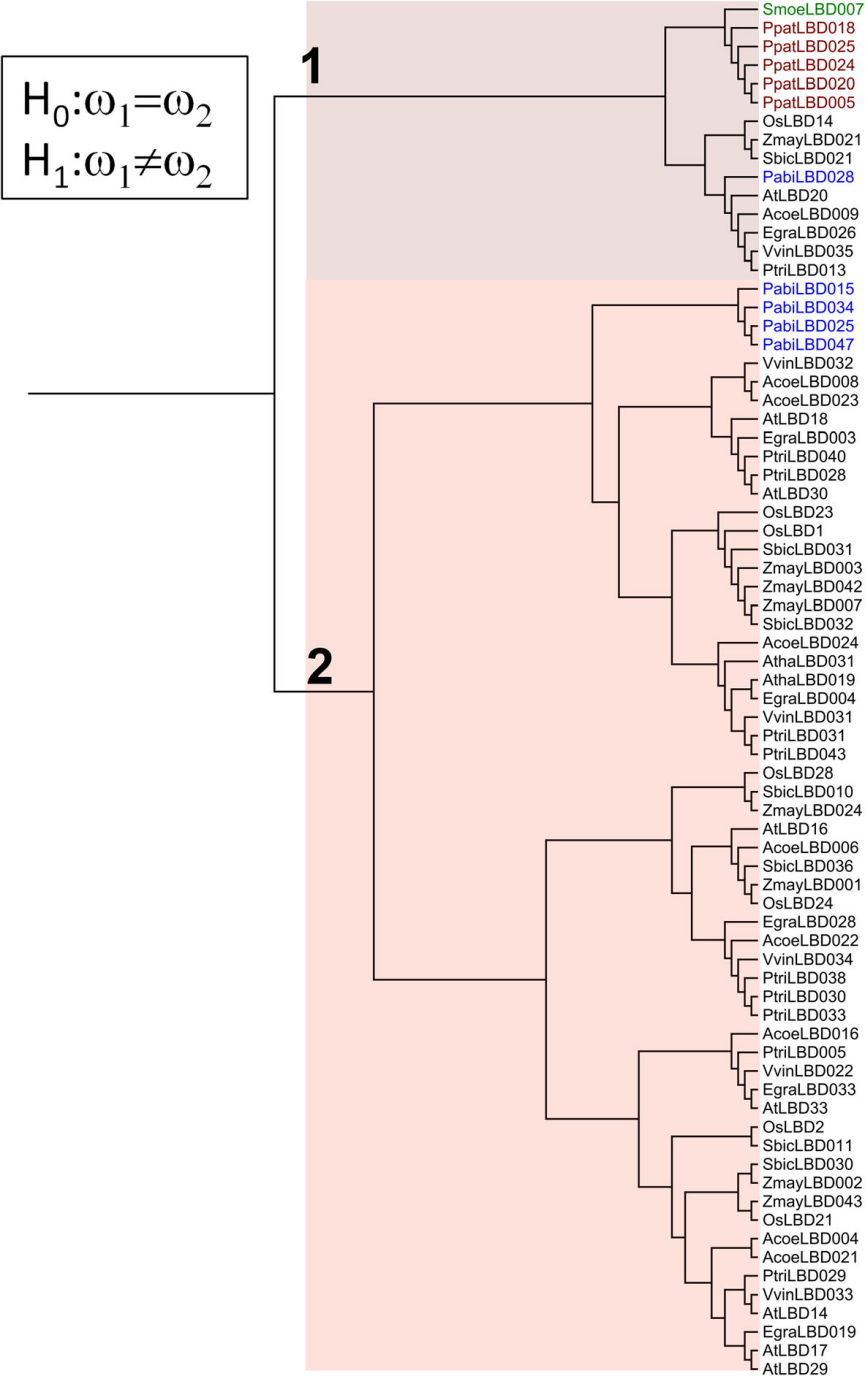
Selection pressure analysis of class IB *LBD* genes. Phylogenetic tree is constructed by maximum likelihood method. Genes of bryophytes, lycophytes, gymnosperms and angiosperms are indicated by red, green, blue and black color, respectively. Differences in selection pressure are modeled by specifying different ω ratios for ancestral lineages and seed plant-specific lineages. Two hypotheses of variable selection pressure are specified as H_0_ and H_1_. H_0_: ω_1_ = ω_2_ = 0.03395, *l* = -8133.64; H_1_: ω_1_ = 0.0220, ω_2_ = 0.0382, *l* = -8129.48; *p*-value = 0.0039 (df = 1)

In seed plants, class IB *LBD* genes display prominent functions in regulating root development [6, 9, 10]. Previous study detected none of class IB *LBD* gene exists in *Selaginella moellendorphii* [1], leading to the assumption that genetic programs of root development in lycophytes are distinctive to the seed plants. In contrast, our method is sufficient to identify a basal-node *LBD* gene in *S. moellendorphii* (*SmoeLBD007*), which clusters with class IB genes with high support (aLRT = 95%) (Fig. 6). This ancient gene lineage is preserved in all of the land plant genomes analyzed, and its direct descendant in *Arabidopsis* is *AtLBD20*, which was demonstrated to participate in pathogen defense [28]. Realtime PCR shows *SmoeLBD007* is mainly expressed in root and leaf tissues (Additional file 6), suggesting it is functional during these tissues development. Meanwhile, phylogenetic analysis identifies two *LBD* gene groups that are specific to seed plants. These gene groups contain some key regulators of lateral root development in angiosperms, including *AtLBD16*, *AtLBD18* and *AtLBD29* in *Arabidopsis*, *OsLBD21/CRL1* in rice and *ZmayLBD002/RTCS* in maize [6, 9, 10]. Selection pressure analysis shows that the seed plant-specific gene lineages exhibit a higher nonsynonymous/synonymous substitution ratio (k_a_/k_s_, *p*-value = 0.0039) (Fig. 6), suggesting they accumulated more nonsynonymous amino acid changes during seed plants evolution. Therefore, although class IB *LBD* genes are present in *S. moellendorphii*, they are extensively duplicated in seed plants, and recruited to root regulations through sequence change and functional specialization.

Class IA is composed of two ancient lineages, namely IA-1 and IA-2, which give rise to *AtLOB* and *AtLBD6/AS2* in *Arabidopsis* respectively (Fig. 4). Consistent with the independent gene ancestry, *AtLOB* and *AtLBD6/AS2* exhibit divergent biological functions even with highly similar amino acid compositions [5]. Class IA-2 experienced a duplication event before angiosperm emergence and generates *AtLBD6/AS2* lineage and *AtLBD10* lineage. Sequence alignment identifies a “SKYQ” motif immediately downstream of the LOB domain in *AtLBD6/AS2* orthologs (Additional file 7). In contrast, *AtLBD10* orthologs contain a totally different sequence featured with “AAYIGP”. This phenomenon suggests protein sequence change is also accompanied with evolution of class IA *LBD* genes in angiosperms.

Class II subfamily contains two *LBD* gene ancestors before seed plant appearance. In *Arabidopsis*, *AtLBD37*, *AtLBD38* and *AtLBD39* are derived from a common ancestor (Fig. 4) and participate in a same biological process to repress anthocyanin biosynthesis and nitrogen responsive genes [8]. Through sequence alignment, we identify a featured pattern of LxLxL motif in these proteins (Additional file 7) which is required to recruit TPL/TPR co-repressors and fulfill transcriptional repression activity [29]. The LxLxL motif is also conserved in class II *LBD* genes of *Physcomitrella*, suggesting the ability to recruit TPL/TPR co-repressors has been acquired by early land plants.

## Conclusions

In this study we present an improved method for *LBD* gene detection, and identify 431 *LBD* genes in 11 high-quality genomes of land plants. Through gene collinearity and phylogenetic analyses, we retrace 7 ancient *LBD* gene lineages in early land plants, which gave rise to 11 ancestor genes for seed plants and 18 ancestor genes for angiosperms through gene duplications. All of the ancient gene lineages are preserved by current genomes of land plant, including the previously controversial class IB gene in *S. moellendorphii*, suggesting *LBD* genes are extremely reluctant to be lost during evolution. On the other hand, whole-genome and dispersed gene duplications, accompanied with frequent protein sequence change and expression pattern alteration, account for the major expansions of *LBD* genes in angiosperms, which illustrates an important scheme for *LBD* gene family diversification.

## Methods

### Sequence retrieval

LBD protein sequences are retrieved from public genome databases, including *Aquilegia coerulea* (JGI v1.1); *Arabidopsis thaliana* (TAIR 10); *Eucalyptus grandis* (JGI v2.0); *Oryza sativa subsp. japonica* (MSU v7.0); *Picea abies* (ConGenIE v1.0); *Physcomitrella patens subsp. patens* (JGI v3.0); *Populus trichocarpa* (JGI v3.0); *Sorghum bicolor* (JGI v2.1); *Selaginella moellendorffii* (JGI v1.0); *Vitis vinifera* (Genoscope 12X); *Zea mays* (MaizeSequence Release 6a). The LOB domain (designated as DUF260 in the PFAM database) is searched to identify putative LBD proteins. Hmmsearch (HMMer package version3.1b1) is used to search the PFAM profile hidden Markov model (pHMM) DUF260.hmm (http://pfam.xfam.org/) [30] against protein sequences from each genome. To ensure the searching reliability, domain hits beyond the gathering threshold (E-value 1e-5) and less than 80% of the coverage are filtered out before downstream analysis. We also remove redundant protein sequences which are alternatively spliced from the same locus. *LBD* genes in *Arabidopsis* and maize were named according to previous studies, while gene sequences from other species were renamed for simplicity (Additional file 1).

### Gene duplication events and collinearity relationship analysis

MCscanX is used to detect gene duplication types and collineartiy relationships [31]. For seven angiosperms (*A. thaliana, E. grandis, P. trichocarpa*, *V. vinifera*, *O. sativa*, *S. bicolor* and *Z. mays*), all annotated proteins in each genome were self-to-self compared by BLASTP (version 2.2.21) program with E-value 1e-10. The top 5 BLASTP hits of each gene were retained for downstream analysis of syntenic regions. The *duplicate_gene_classifier* program incorporated in MCscanX is used to identify different duplication types in a genome: whole genome duplication (collinear genes in syntenic blocks), tandem duplication (consecutive repeat genes), proximal duplication (genes spanning less than 20 genes in nearby chromosomal region) and dispersed duplication (other modes than whole genome, tandem and proximal duplications).

MCscanX is used to detect collinear blocks within dicot or monocot separately. Protein sequences of dicots (*A. thaliana, E. grandis, P. trichocarpa* and *V. vinifera*) or monocots (*O. sativa*, *S. bicolor* and *Z. mays*) were pooled independently to conduct self-self comparison by BLASTP (version 2.2.21) program with same parameters as described above (‘-e 1e-10 -b5 -v5 -m8’). PERL script *detect_collinearity_within_gene_families.pl* incorporated in MCscanX is used to detect collinearity within *LBD* gene family. *LBD* genes derived from collinear blocks are recursively merged into collinear groups using custom PERL script.

### Phylogenetic analysis

To generate phylogenetic tree of *LBD* genes in land plants, the protein sequences of *LBD* genes are aligned to PFAM profile hidden Markov models of the LOB domain (pHMM DUF260.hmm) using HMMalign (HMMer package version 3.1b1) [30]. Terminal tails of non-aligned residues are trimmed using parameter ‘--trim’ and only unambiguous alignment of each sequence is subjected for subsequent phylogenetic analyses. The Jones, Taylor, and Thorton (JTT) model is selected as the best-fitting amino acid substitution model according to the Akaike information criterion (AIC) and the Bayesian information criterion (BIC) scores estimated by ProtTest (v3.3) [32]. The maximum likelihood (ML) analysis is performed by the program PhyML (version 3.1) using the JTT model of amino acid substitution, four gamma-distributed rate categories and the Shimodaira-Hasegawa-like approximate likelihood-ratio test (SH-aLRT) [33]. Reliability of the internal branches is evaluated based on SH-aLRT supports. The tree is started from BIONJ tree and the topology of the tree is improved by subtree pruning and regrafting (SPR) method from 10 random starting trees. The output tree is visualized in the program Figtree (http://tree.bio.ed.ac.uk/software/figtree/).

To analyze class IA *LBD* genes, complete protein sequences were aligned with MUSCLE (v3.7) with default parameters [34]. Multiple sequence alignments were trimmed by removing poorly aligned regions using TRIMAL (v1.4.rev15) with the option ‘-automated1’ [35]. The ML analysis is performed by the program PhyML (version 3.1) using the JTT model of amino acid substitution, four gamma-distributed rate categories and SH-aLRT test. The tree is started from BIONJ tree and the topology of the tree is improved by SPR method from 10 random starting trees.

### Sequence logos of LBD proteins

For each LBD class, MUSCLE (v3.7) was used to align the complete protein sequences with default parameters [34]. Nucleotide conservation was hereafter analyzed and shown with WebLogo (v3.3) (http://weblogo.threeplusone.com/).

### Selection pressure analysis for class IB LBD genes

Selection pressure analysis is measured by ω parameter in PAML (v4.6) package [36], which is the nonsynonymous/synonymous substitution rate ratio. Two hypotheses of variable selection pressure are modeled as H_0_ and H_1_. While the null model (H_0_) assigns only one ω for the whole tree, the branch model (H_1_) assigns two independent ω values for two branches. Codeml program is used to obtain the log likelihood by performing multiple analyses with a range of initial values for the ω parameter. Significant likelihood ratio tests (LRTs) is used to conduct the significance of difference between two models by chi2 program.

## Additional files

Additional file 1:
*LBD* genes identified in this study. (PDF 19 kb)
Additional file 2: Collinear *LBD* gene groups in dicots and monocots. (PDF 50 kb)
Additional file 3: ML tree of *LBD* genes in land plants. (PDF 74 kb)
Additional file 4: ML tree of class IA *LBD* genes. (TIF 1275 kb)
Additional file 5: Sequence logo of LBD proteins in each class. (TIF 2487 kb)
Additional file 6: Expression of class IB *LBD* gene in *S. moellendorphii*. (TIF 381 kb)
Additional file 7: Representative motifs for class IA and class II genes. (TIF 4927 kb)
