# Supplementary material for: Decipher the ancestry of the plant-specific LBD gene family
Source: BMC Genomics. 2017 Jan 25;18(Suppl 1):951. doi: 10.1186/s12864-016-3264-3 (PMC5310275; doi:10.1186/s12864-016-3264-3)
Supplement: Additional file 1: — LBD genes identified in this study. (PDF 19 kb) [file 12864_2016_3264_MOESM1_ESM.pdf]

Additional file 1. LBD genes identified in this study.

Gene Name	Abbreviation	Species
Aquca_005_00169	AcoeLBD001	*Aquilegia coerulea*
Aquca_020_00437	AcoeLBD002	*Aquilegia coerulea*
Aquca_045_00193	AcoeLBD003	*Aquilegia coerulea*
Aquca_017_00693	AcoeLBD004	*Aquilegia coerulea*
Aquca_017_00034	AcoeLBD005	*Aquilegia coerulea*
Aquca_017_00683	AcoeLBD006	*Aquilegia coerulea*
Aquca_042_00143	AcoeLBD007	*Aquilegia coerulea*
Aquca_009_00958	AcoeLBD008	*Aquilegia coerulea*
Aquca_009_00560	AcoeLBD009	*Aquilegia coerulea*
Aquca_030_00227	AcoeLBD010	*Aquilegia coerulea*
Aquca_011_00559	AcoeLBD011	*Aquilegia coerulea*
Aquca_018_00023	AcoeLBD012	*Aquilegia coerulea*
Aquca_118_00035	AcoeLBD013	*Aquilegia coerulea*
Aquca_027_00112	AcoeLBD014	*Aquilegia coerulea*
Aquca_007_00083	AcoeLBD015	*Aquilegia coerulea*
Aquca_003_00139	AcoeLBD016	*Aquilegia coerulea*
Aquca_058_00006	AcoeLBD017	*Aquilegia coerulea*
Aquca_002_00445	AcoeLBD018	*Aquilegia coerulea*
Aquca_036_00001	AcoeLBD019	*Aquilegia coerulea*
Aquca_026_00293	AcoeLBD020	*Aquilegia coerulea*
Aquca_034_00400	AcoeLBD021	*Aquilegia coerulea*
Aquca_034_00401	AcoeLBD022	*Aquilegia coerulea*
Aquca_013_00578	AcoeLBD023	*Aquilegia coerulea*
Aquca_013_00580	AcoeLBD024	*Aquilegia coerulea*
Aquca_061_00084	AcoeLBD025	*Aquilegia coerulea*
Aquca_025_00139	AcoeLBD026	*Aquilegia coerulea*
AT2G19820	AtLBD9	*Arabidopsis thaliana*
AT2G42440	AtLBD17	*Arabidopsis thaliana*
AT2G30130	AtLBD12	*Arabidopsis thaliana*
AT2G19510	AtLBD8	*Arabidopsis thaliana*
AT2G28500	AtLBD11	*Arabidopsis thaliana*
AT2G31310	AtLBD14	*Arabidopsis thaliana*
AT2G42430	AtLBD16	*Arabidopsis thaliana*
AT2G30340	AtLBD13	*Arabidopsis thaliana*
AT2G45410	AtLBD19	*Arabidopsis thaliana*
AT2G40470	AtLBD15	*Arabidopsis thaliana*
AT2G45420	AtLBD18	*Arabidopsis thaliana*
AT2G23660	AtLBD10	*Arabidopsis thaliana*
AT4G22700	AtLBD32	*Arabidopsis thaliana*
AT4G00210	AtLBD31	*Arabidopsis thaliana*
AT4G00220	AtLBD30	*Arabidopsis thaliana*
AT4G37540	AtLBD39	*Arabidopsis thaliana*
AT1G72980	AtLBD7	*Arabidopsis thaliana*
AT1G06280	AtLBD2	*Arabidopsis thaliana*

Gene Name	Abbreviation	Species
AT1G31320	AtLBD4	*Arabidopsis thaliana*
AT1G67100	AtLBD40	*Arabidopsis thaliana*
AT1G16530	AtLBD3	*Arabidopsis thaliana*
AT1G68510	AtLBD42	*Arabidopsis thaliana*
AT1G65620	AtLBD6	*Arabidopsis thaliana*
AT1G07900	AtLBD1	*Arabidopsis thaliana*
AT1G36000	AtLBD5	*Arabidopsis thaliana*
AT3G26660	AtLBD24	*Arabidopsis thaliana*
AT3G47870	AtLBD27	*Arabidopsis thaliana*
AT3G13850	AtLBD22	*Arabidopsis thaliana*
AT3G50510	AtLBD28	*Arabidopsis thaliana*
AT3G02550	AtLBD41	*Arabidopsis thaliana*
AT3G58190	AtLBD29	*Arabidopsis thaliana*
AT3G03760	AtLBD20	*Arabidopsis thaliana*
AT3G27940	AtLBD26	*Arabidopsis thaliana*
AT3G49940	AtLBD38	*Arabidopsis thaliana*
AT3G11090	AtLBD21	*Arabidopsis thaliana*
AT3G27650	AtLBD25	*Arabidopsis thaliana*
AT3G26620	AtLBD23	*Arabidopsis thaliana*
AT5G35900	AtLBD35	*Arabidopsis thaliana*
AT5G15060	AtLBD34	*Arabidopsis thaliana*
AT5G67420	AtLBD37	*Arabidopsis thaliana*
AT5G06080	AtLBD33	*Arabidopsis thaliana*
AT5G63090	AtLBDLOB	*Arabidopsis thaliana*
AT5G66870	AtLBD36	*Arabidopsis thaliana*
Eucgr.E04355	EgraLBD001	*Eucalyptus grandis*
Eucgr.E02016	EgraLBD002	*Eucalyptus grandis*
Eucgr.E01414	EgraLBD003	*Eucalyptus grandis*
Eucgr.E01415	EgraLBD004	*Eucalyptus grandis*
Eucgr.E02018	EgraLBD005	*Eucalyptus grandis*
Eucgr.L00754	EgraLBD006	*Eucalyptus grandis*
Eucgr.J00175	EgraLBD007	*Eucalyptus grandis*
Eucgr.J01501	EgraLBD008	*Eucalyptus grandis*
Eucgr.J03160	EgraLBD009	*Eucalyptus grandis*
Eucgr.J01123	EgraLBD010	*Eucalyptus grandis*
Eucgr.J02237	EgraLBD011	*Eucalyptus grandis*
Eucgr.J02250	EgraLBD012	*Eucalyptus grandis*
Eucgr.C02148	EgraLBD013	*Eucalyptus grandis*
Eucgr.H02293	EgraLBD014	*Eucalyptus grandis*
Eucgr.A02814	EgraLBD015	*Eucalyptus grandis*
Eucgr.F04424	EgraLBD016	*Eucalyptus grandis*
Eucgr.F00115	EgraLBD017	*Eucalyptus grandis*
Eucgr.F02492	EgraLBD018	*Eucalyptus grandis*
Eucgr.B01529	EgraLBD019	*Eucalyptus grandis*
Eucgr.B03016	EgraLBD020	*Eucalyptus grandis*

Gene Name	Abbreviation	Species
Eucgr.B04019	EgraLBD021	*Eucalyptus grandis*
Eucgr.B02465	EgraLBD022	*Eucalyptus grandis*
Eucgr.B03991	EgraLBD023	*Eucalyptus grandis*
Eucgr.I01750	EgraLBD024	*Eucalyptus grandis*
Eucgr.I01908	EgraLBD025	*Eucalyptus grandis*
Eucgr.K01522	EgraLBD026	*Eucalyptus grandis*
Eucgr.K02222	EgraLBD027	*Eucalyptus grandis*
Eucgr.K03284	EgraLBD028	*Eucalyptus grandis*
Eucgr.K02809	EgraLBD029	*Eucalyptus grandis*
Eucgr.G02321	EgraLBD030	*Eucalyptus grandis*
Eucgr.G03089	EgraLBD031	*Eucalyptus grandis*
Eucgr.G03078	EgraLBD032	*Eucalyptus grandis*
Eucgr.G02955	EgraLBD033	*Eucalyptus grandis*
Eucgr.G03121	EgraLBD034	*Eucalyptus grandis*
Eucgr.G01261	EgraLBD035	*Eucalyptus grandis*
LOC_Os10g07510	OsLBD1	*Oryza sativa*
LOC_Os08g44940	OsLBD2	*Oryza sativa*
LOC_Os08g06659	OsLBD3	*Oryza sativa*
LOC_Os08g31080	OsLBD4	*Oryza sativa*
LOC_Os07g40000	OsLBD5	*Oryza sativa*
LOC_Os01g39160	OsLBD6	*Oryza sativa*
LOC_Os01g14030	OsLBD7	*Oryza sativa*
LOC_Os01g39220	OsLBD8	*Oryza sativa*
LOC_Os01g39150	OsLBD9	*Oryza sativa*
LOC_Os01g39040	OsLBD10	*Oryza sativa*
LOC_Os01g66590	OsLBD11	*Oryza sativa*
LOC_Os01g60960	OsLBD12	*Oryza sativa*
LOC_Os01g07480	OsLBD13	*Oryza sativa*
LOC_Os01g56530	OsLBD14	*Oryza sativa*
LOC_Os01g39070	OsLBD15	*Oryza sativa*
LOC_Os01g39180	OsLBD16	*Oryza sativa*
LOC_Os01g32770	OsLBD17	*Oryza sativa*
LOC_Os01g03890	OsLBD18	*Oryza sativa*
LOC_Os03g33090	OsLBD19	*Oryza sativa*
LOC_Os03g17810	OsLBD20	*Oryza sativa*
LOC_Os03g05510	OsLBD21	*Oryza sativa*
LOC_Os03g41330	OsLBD22	*Oryza sativa*
LOC_Os03g14270	OsLBD23	*Oryza sativa*
LOC_Os03g05500	OsLBD24	*Oryza sativa*
LOC_Os03g57670	OsLBD25	*Oryza sativa*
LOC_Os03g45750	OsLBD26	*Oryza sativa*
LOC_Os03g41600	OsLBD27	*Oryza sativa*
LOC_Os02g57490	OsLBD28	*Oryza sativa*
LOC_Os09g19950	OsLBD29	*Oryza sativa*
LOC_Os12g01550	OsLBD30	*Oryza sativa*

Gene Name	Abbreviation	Species
LOC_Os05g34450	OsLBD31	*Oryza sativa*
LOC_Os05g07270	OsLBD32	*Oryza sativa*
LOC_Os05g27980	OsLBD33	*Oryza sativa*
LOC_Os11g01550	OsLBD34	*Oryza sativa*
MA_127139g0010	PabiLBD001	*Picea abies*
MA_19934g0010	PabiLBD002	*Picea abies*
MA_161608g0010	PabiLBD003	*Picea abies*
MA_7393g0010	PabiLBD004	*Picea abies*
MA_39832g0010	PabiLBD005	*Picea abies*
MA_61242g0010	PabiLBD006	*Picea abies*
MA_75731g0010	PabiLBD007	*Picea abies*
MA_16891g0010	PabiLBD008	*Picea abies*
MA_16891g0020	PabiLBD009	*Picea abies*
MA_9924708g0010	PabiLBD010	*Picea abies*
MA_10434782g0020	PabiLBD011	*Picea abies*
MA_63523g0010	PabiLBD012	*Picea abies*
MA_85817g0010	PabiLBD013	*Picea abies*
MA_85817g0020	PabiLBD014	*Picea abies*
MA_962403g0010	PabiLBD015	*Picea abies*
MA_223267g0010	PabiLBD016	*Picea abies*
MA_234967g0010	PabiLBD017	*Picea abies*
MA_84752g0010	PabiLBD018	*Picea abies*
MA_361391g0010	PabiLBD019	*Picea abies*
MA_170369g0010	PabiLBD020	*Picea abies*
MA_927067g0010	PabiLBD021	*Picea abies*
MA_88711g0010	PabiLBD022	*Picea abies*
MA_66621g0010	PabiLBD023	*Picea abies*
MA_109483g0010	PabiLBD024	*Picea abies*
MA_8060019g0010	PabiLBD025	*Picea abies*
MA_79692g0010	PabiLBD026	*Picea abies*
MA_19148g0010	PabiLBD027	*Picea abies*
MA_126761g0010	PabiLBD028	*Picea abies*
MA_174550g0010	PabiLBD029	*Picea abies*
MA_70989g0010	PabiLBD030	*Picea abies*
MA_55357g0010	PabiLBD031	*Picea abies*
MA_170661g0010	PabiLBD032	*Picea abies*
MA_80122g0010	PabiLBD033	*Picea abies*
MA_101817g0010	PabiLBD034	*Picea abies*
MA_185511g0010	PabiLBD035	*Picea abies*
MA_10429262g0010	PabiLBD036	*Picea abies*
MA_131805g0010	PabiLBD037	*Picea abies*
MA_66501g0010	PabiLBD038	*Picea abies*
MA_50096g0010	PabiLBD039	*Picea abies*
MA_19975g0010	PabiLBD040	*Picea abies*
MA_20012g0010	PabiLBD041	*Picea abies*

Gene Name	Abbreviation	Species
MA_11285g0020	PabiLBD042	*Picea abies*
MA_9412105g0010	PabiLBD043	*Picea abies*
MA_159982g0010	PabiLBD044	*Picea abies*
MA_328535g0010	PabiLBD045	*Picea abies*
MA_91369g0010	PabiLBD046	*Picea abies*
MA_86300g0010	PabiLBD047	*Picea abies*
MA_113501g0010	PabiLBD048	*Picea abies*
MA_292200g0010	PabiLBD049	*Picea abies*
MA_445236g0010	PabiLBD050	*Picea abies*
MA_78245g0010	PabiLBD051	*Picea abies*
MA_13024g0010	PabiLBD052	*Picea abies*
MA_77200g0010	PabiLBD053	*Picea abies*
MA_5258645g0010	PabiLBD054	*Picea abies*
MA_9033878g0010	PabiLBD055	*Picea abies*
MA_10056514g0010	PabiLBD056	*Picea abies*
MA_10432553g0010	PabiLBD057	*Picea abies*
MA_35037g0010	PabiLBD058	*Picea abies*
MA_3123g0010	PabiLBD059	*Picea abies*
MA_451290g0010	PabiLBD060	*Picea abies*
MA_16646g0010	PabiLBD061	*Picea abies*
MA_290245g0010	PabiLBD062	*Picea abies*
MA_129671g0010	PabiLBD063	*Picea abies*
MA_136689g0010	PabiLBD064	*Picea abies*
MA_122595g0010	PabiLBD065	*Picea abies*
MA_97411g0010	PabiLBD066	*Picea abies*
MA_10434193g0010	PabiLBD067	*Picea abies*
MA_172003g0010	PabiLBD068	*Picea abies*
MA_9839150g0010	PabiLBD069	*Picea abies*
MA_37490g0010	PabiLBD070	*Picea abies*
MA_902082g0010	PabiLBD071	*Picea abies*
Phpat.022G022200	PpatLBD001	*Physcomitrella patens*
Phpat.022G059900	PpatLBD002	*Physcomitrella patens*
Phpat.019G066500	PpatLBD003	*Physcomitrella patens*
Phpat.019G066100	PpatLBD004	*Physcomitrella patens*
Phpat.003G110400	PpatLBD005	*Physcomitrella patens*
Phpat.020G032200	PpatLBD006	*Physcomitrella patens*
Phpat.020G041900	PpatLBD007	*Physcomitrella patens*
Phpat.020G038800	PpatLBD008	*Physcomitrella patens*
Phpat.020G027600	PpatLBD009	*Physcomitrella patens*
Phpat.023G021400	PpatLBD010	*Physcomitrella patens*
Phpat.023G050100	PpatLBD011	*Physcomitrella patens*
Phpat.024G023100	PpatLBD012	*Physcomitrella patens*
Phpat.027G014200	PpatLBD013	*Physcomitrella patens*
Phpat.009G085600	PpatLBD014	*Physcomitrella patens*
Phpat.009G088700	PpatLBD015	*Physcomitrella patens*

Gene Name	Abbreviation	Species
Phpat.021G057100	PpatLBD016	*Physcomitrella patens*
Phpat.005G025200	PpatLBD017	*Physcomitrella patens*
Phpat.011G041100	PpatLBD018	*Physcomitrella patens*
Phpat.014G095400	PpatLBD019	*Physcomitrella patens*
Phpat.008G019000	PpatLBD020	*Physcomitrella patens*
Phpat.008G080600	PpatLBD021	*Physcomitrella patens*
Phpat.008G050800	PpatLBD022	*Physcomitrella patens*
Phpat.004G071300	PpatLBD023	*Physcomitrella patens*
Phpat.004G034100	PpatLBD024	*Physcomitrella patens*
Phpat.007G042000	PpatLBD025	*Physcomitrella patens*
Phpat.015G084700	PpatLBD026	*Physcomitrella patens*
Phpat.012G019600	PpatLBD027	*Physcomitrella patens*
Phpat.018G031700	PpatLBD028	*Physcomitrella patens*
Potri.010G125000	PtriLBD001	*Populus trichocarpa*
Potri.010G250700	PtriLBD002	*Populus trichocarpa*
Potri.010G186000	PtriLBD003	*Populus trichocarpa*
Potri.010G177100	PtriLBD004	*Populus trichocarpa*
Potri.010G200400	PtriLBD005	*Populus trichocarpa*
Potri.010G184400	PtriLBD006	*Populus trichocarpa*
Potri.010G217700	PtriLBD007	*Populus trichocarpa*
Potri.017G114500	PtriLBD008	*Populus trichocarpa*
Potri.009G089600	PtriLBD009	*Populus trichocarpa*
Potri.009G076900	PtriLBD010	*Populus trichocarpa*
Potri.T062400	PtriLBD011	*Populus trichocarpa*
Potri.004G100100	PtriLBD012	*Populus trichocarpa*
Potri.013G064500	PtriLBD013	*Populus trichocarpa*
Potri.013G156200	PtriLBD014	*Populus trichocarpa*
Potri.013G081200	PtriLBD015	*Populus trichocarpa*
Potri.013G123900	PtriLBD016	*Populus trichocarpa*
Potri.003G037300	PtriLBD017	*Populus trichocarpa*
Potri.003G149000	PtriLBD018	*Populus trichocarpa*
Potri.003G039700	PtriLBD019	*Populus trichocarpa*
Potri.006G228300	PtriLBD020	*Populus trichocarpa*
Potri.018G052700	PtriLBD021	*Populus trichocarpa*
Potri.007G066700	PtriLBD022	*Populus trichocarpa*
Potri.007G053600	PtriLBD023	*Populus trichocarpa*
Potri.007G039500	PtriLBD024	*Populus trichocarpa*
Potri.015G082200	PtriLBD025	*Populus trichocarpa*
Potri.015G135900	PtriLBD026	*Populus trichocarpa*
Potri.015G066700	PtriLBD027	*Populus trichocarpa*
Potri.002G149000	PtriLBD028	*Populus trichocarpa*
Potri.002G041100	PtriLBD029	*Populus trichocarpa*
Potri.002G041200	PtriLBD030	*Populus trichocarpa*
Potri.002G148900	PtriLBD031	*Populus trichocarpa*
Potri.002G119400	PtriLBD032	*Populus trichocarpa*

Gene Name	Abbreviation	Species
Potri.T078600	PtriLBD033	*Populus trichocarpa*
Potri.019G127300	PtriLBD034	*Populus trichocarpa*
Potri.019G092200	PtriLBD035	*Populus trichocarpa*
Potri.005G145500	PtriLBD036	*Populus trichocarpa*
Potri.005G134900	PtriLBD037	*Populus trichocarpa*
Potri.005G221900	PtriLBD038	*Populus trichocarpa*
Potri.005G097800	PtriLBD039	*Populus trichocarpa*
Potri.014G070400	PtriLBD040	*Populus trichocarpa*
Potri.014G017400	PtriLBD041	*Populus trichocarpa*
Potri.014G167100	PtriLBD042	*Populus trichocarpa*
Potri.014G070300	PtriLBD043	*Populus trichocarpa*
Potri.008G120600	PtriLBD044	*Populus trichocarpa*
Potri.008G043900	PtriLBD045	*Populus trichocarpa*
Potri.008G072800	PtriLBD046	*Populus trichocarpa*
Potri.008G071500	PtriLBD047	*Populus trichocarpa*
Potri.008G079800	PtriLBD048	*Populus trichocarpa*
Potri.001G081400	PtriLBD049	*Populus trichocarpa*
Potri.001G196400	PtriLBD050	*Populus trichocarpa*
Potri.001G295700	PtriLBD051	*Populus trichocarpa*
Potri.001G281600	PtriLBD052	*Populus trichocarpa*
Potri.001G345700	PtriLBD053	*Populus trichocarpa*
Potri.012G056800	PtriLBD054	*Populus trichocarpa*
Potri.012G133700	PtriLBD055	*Populus trichocarpa*
Potri.012G083500	PtriLBD056	*Populus trichocarpa*
Potri.012G072000	PtriLBD057	*Populus trichocarpa*
Sobic.010G259400	SbicLBD001	*Sorghum bicolor*
Sobic.010G037700	SbicLBD002	*Sorghum bicolor*
Sobic.009G258300	SbicLBD003	*Sorghum bicolor*
Sobic.009G058600	SbicLBD004	*Sorghum bicolor*
Sobic.009G199400	SbicLBD005	*Sorghum bicolor*
Sobic.009G199300	SbicLBD006	*Sorghum bicolor*
Sobic.009G102400	SbicLBD007	*Sorghum bicolor*
Sobic.009G025900	SbicLBD008	*Sorghum bicolor*
Sobic.009G138500	SbicLBD009	*Sorghum bicolor*
Sobic.004G348800	SbicLBD010	*Sorghum bicolor*
Sobic.007G165100	SbicLBD011	*Sorghum bicolor*
Sobic.007G200400	SbicLBD012	*Sorghum bicolor*
Sobic.007G122600	SbicLBD013	*Sorghum bicolor*
Sobic.002G182700	SbicLBD014	*Sorghum bicolor*
Sobic.002G040000	SbicLBD015	*Sorghum bicolor*
Sobic.002G359700	SbicLBD016	*Sorghum bicolor*
Sobic.003G192800	SbicLBD017	*Sorghum bicolor*
Sobic.003G340100	SbicLBD018	*Sorghum bicolor*
Sobic.003G052900	SbicLBD019	*Sorghum bicolor*
Sobic.003G383700	SbicLBD020	*Sorghum bicolor*

Gene Name	Abbreviation	Species
Sobic.003G311100	SbicLBD021	*Sorghum bicolor*
Sobic.003G086600	SbicLBD022	*Sorghum bicolor*
Sobic.005G004700	SbicLBD023	*Sorghum bicolor*
Sobic.008G190800	SbicLBD024	*Sorghum bicolor*
Sobic.008G005000	SbicLBD025	*Sorghum bicolor*
Sobic.001G169200	SbicLBD026	*Sorghum bicolor*
Sobic.001G328500	SbicLBD027	*Sorghum bicolor*
Sobic.001G147000	SbicLBD028	*Sorghum bicolor*
Sobic.001G409200	SbicLBD029	*Sorghum bicolor*
Sobic.001G503000	SbicLBD030	*Sorghum bicolor*
Sobic.001G437300	SbicLBD031	*Sorghum bicolor*
Sobic.001G261700	SbicLBD032	*Sorghum bicolor*
Sobic.001G167600	SbicLBD033	*Sorghum bicolor*
Sobic.001G338600	SbicLBD034	*Sorghum bicolor*
Sobic.001G062800	SbicLBD035	*Sorghum bicolor*
Sobic.001G503100	SbicLBD036	*Sorghum bicolor*
27657	SmoeLBD001	*Selaginella moellendo*
59179	SmoeLBD002	*Selaginella moellendo*
111283	SmoeLBD003	*Selaginella moellendo*
37169	SmoeLBD004	*Selaginella moellendo*
111494	SmoeLBD005	*Selaginella moellendo*
58936	SmoeLBD006	*Selaginella moellendo*
59642	SmoeLBD007	*Selaginella moellendo*
59650	SmoeLBD008	*Selaginella moellendo*
105005	SmoeLBD009	*Selaginella moellendo*
37570	SmoeLBD010	*Selaginella moellendo*
69267	SmoeLBD011	*Selaginella moellendo*
61520	SmoeLBD012	*Selaginella moellendo*
68988	SmoeLBD013	*Selaginella moellendo*
69621	SmoeLBD014	*Selaginella moellendo*
49084	SmoeLBD015	*Selaginella moellendo*
GSVIVT01000141001	VvinLBD001	*Vitis vinifera*
GSVIVT01003546001	VvinLBD002	*Vitis vinifera*
GSVIVT01003547001	VvinLBD003	*Vitis vinifera*
GSVIVT01003548001	VvinLBD004	*Vitis vinifera*
GSVIVT01004323001	VvinLBD005	*Vitis vinifera*
GSVIVT01006269001	VvinLBD006	*Vitis vinifera*
GSVIVT01008080001	VvinLBD007	*Vitis vinifera*
GSVIVT01008284001	VvinLBD008	*Vitis vinifera*
GSVIVT01009360001	VvinLBD009	*Vitis vinifera*
GSVIVT01010625001	VvinLBD010	*Vitis vinifera*
GSVIVT01011895001	VvinLBD011	*Vitis vinifera*
GSVIVT01011896001	VvinLBD012	*Vitis vinifera*
GSVIVT01013631001	VvinLBD013	*Vitis vinifera*
GSVIVT01016327001	VvinLBD014	*Vitis vinifera*

Gene Name	Abbreviation	Species
GSVIVT01016328001	VvinLBD015	*Vitis vinifera*
GSVIVT01016329001	VvinLBD016	*Vitis vinifera*
GSVIVT01016330001	VvinLBD017	*Vitis vinifera*
GSVIVT01016332001	VvinLBD018	*Vitis vinifera*
GSVIVT01016333001	VvinLBD019	*Vitis vinifera*
GSVIVT01016334001	VvinLBD020	*Vitis vinifera*
GSVIVT01016335001	VvinLBD021	*Vitis vinifera*
GSVIVT01016500001	VvinLBD022	*Vitis vinifera*
GSVIVT01018486001	VvinLBD023	*Vitis vinifera*
GSVIVT01020353001	VvinLBD024	*Vitis vinifera*
GSVIVT01024592001	VvinLBD025	*Vitis vinifera*
GSVIVT01024662001	VvinLBD026	*Vitis vinifera*
GSVIVT01025126001	VvinLBD027	*Vitis vinifera*
GSVIVT01025128001	VvinLBD028	*Vitis vinifera*
GSVIVT01027172001	VvinLBD029	*Vitis vinifera*
GSVIVT01027173001	VvinLBD030	*Vitis vinifera*
GSVIVT01027620001	VvinLBD031	*Vitis vinifera*
GSVIVT01027621001	VvinLBD032	*Vitis vinifera*
GSVIVT01028294001	VvinLBD033	*Vitis vinifera*
GSVIVT01028295001	VvinLBD034	*Vitis vinifera*
GSVIVT01029979001	VvinLBD035	*Vitis vinifera*
GSVIVT01031035001	VvinLBD036	*Vitis vinifera*
GSVIVT01032415001	VvinLBD037	*Vitis vinifera*
GSVIVT01032592001	VvinLBD038	*Vitis vinifera*
GSVIVT01032714001	VvinLBD039	*Vitis vinifera*
GSVIVT01032752001	VvinLBD040	*Vitis vinifera*
GSVIVT01032779001	VvinLBD041	*Vitis vinifera*
GSVIVT01037853001	VvinLBD042	*Vitis vinifera*
AC207888.3_FGP009	ZmayLBD015	*Zea mays*
GRMZM2G177110	ZmayLBD014	*Zea mays*
AC214648.3_FGP005	ZmayLBD039	*Zea mays*
AC234149.1_FGP002	ZmayLBD037	*Zea mays*
GRMZM2G073044	ZmayLBD038	*Zea mays*
GRMZM2G133806	ZmayLBD040	*Zea mays*
GRMZM2G096064	ZmayLBD036	*Zea mays*
GRMZM2G362627	ZmayLBD027	*Zea mays*
GRMZM2G079185	ZmayLBD026	*Zea mays*
GRMZM2G704330	ZmayLBD035	*Zea mays*
GRMZM2G092483	ZmayLBD001	*Zea mays*
GRMZM2G110913	ZmayLBD003	*Zea mays*
GRMZM2G447176	ZmayLBD012	*Zea mays*
AC217910.3_FGP006	ZmayLBD009	*Zea mays*
GRMZM2G044902	ZmayLBD006	*Zea mays*
GRMZM2G092542	ZmayLBD002	*Zea mays*
GRMZM2G076327	ZmayLBD007	*Zea mays*

Gene Name	Abbreviation	Species
GRMZM2G132667	ZmayLBD008	*Zea mays*
GRMZM2G060544	ZmayLBD005	*Zea mays*
GRMZM2G021095	ZmayLBD004	*Zea mays*
GRMZM2G011385	ZmayLBD013	*Zea mays*
GRMZM2G017319	ZmayLBD011	*Zea mays*
GRMZM2G154320	ZmayLBD010	*Zea mays*
GRMZM2G121487	ZmayLBD033	*Zea mays*
GRMZM2G180319	ZmayLBD030	*Zea mays*
GRMZM2G386674	ZmayLBD034	*Zea mays*
GRMZM2G044150	ZmayLBD032	*Zea mays*
GRMZM2G386095	ZmayLBD031	*Zea mays*
GRMZM2G165805	ZmayLBD029	*Zea mays*
GRMZM2G092517	ZmayLBD020	*Zea mays*
AC233943.1_FGP002	ZmayLBD016	*Zea mays*
GRMZM2G025758	ZmayLBD021	*Zea mays*
GRMZM2G132693	ZmayLBD017	*Zea mays*
AC218973.3_FGP003	ZmayLBD018	*Zea mays*
GRMZM2G118250	ZmayLBD019	*Zea mays*
GRMZM2G145568	ZmayLBD041	*Zea mays*
AC149818.2_FGP009	ZmayLBD043	*Zea mays*
GRMZM2G150594	ZmayLBD042	*Zea mays*
GRMZM2G075499	ZmayLBD024	*Zea mays*
GRMZM2G079768	ZmayLBD023	*Zea mays*
GRMZM2G025989	ZmayLBD025	*Zea mays*
GRMZM2G095982	ZmayLBD022	*Zea mays*
GRMZM5G868471	ZmayLBD044	*Zea mays*
GRMZM5G873586	ZmayLBD045	*Zea mays*
