# Supplementary material for: Decipher the ancestry of the plant-specific LBD gene family
Source: BMC Genomics. 2017 Jan 25;18(Suppl 1):951. doi: 10.1186/s12864-016-3264-3 (PMC5310275; doi:10.1186/s12864-016-3264-3)
Supplement: Additional file 2: — Collinear LBD gene groups in dicots and monocots. (PDF 50 kb) [file 12864_2016_3264_MOESM2_ESM.pdf]

Additional file 2. Collinear LBD groups in dicots and monocots.

Group	Class	LBD gene
dicot1	II	AtLBD040 AtLBD042 AtLBD041 EgraLBD020 EgraLBD018 EgraLBD030 EgraLBD008 EgraLBD027 VvinLBD008 VvinLBD011 VvinLBD012 VvinLBD036 PtriLBD012 PtriLBD044 PtriLBD001 PtriLBD054 PtriLBD008
dicot2	II	AtLBD038 AtLBD039 AtLBD037 EgraLBD017 EgraLBD014 VvinLBD001 VvinLBD009 PtriLBD051 PtriLBD032 PtriLBD036 PtriLBD023 PtriLBD009 PtriLBD041
dicot3	IB	AtLBD019 AtLBD018 AtLBD031 AtLBD030 EgraLBD003 EgraLBD004 VvinLBD031 VvinLBD032 PtriLBD031 PtriLBD028 PtriLBD043 PtriLBD040
dicot4	IC1/D	AtLBD012 EgraLBD023 EgraLBD031 EgraLBD007 VvinLBD026 VvinLBD040 PtriLBD052 PtriLBD046 PtriLBD010 PtriLBD006
dicot5	IA	AtLBD025 AtLBDLOB EgraLBD024 EgraLBD011 EgraLBD029 VvinLBD037 PtriLBD053 PtriLBD056 PtriLBD025
dicot6	IC1/D	AtLBD001 AtLBD011 EgraLBD022 EgraLBD010 VvinLBD021 VvinLBD027 VvinLBD028 PtriLBD045 PtriLBD007
dicot7	IC2	AtLBD013 AtLBD015 EgraLBD021 EgraLBD034 VvinLBD025 VvinLBD039 PtriLBD014 PtriLBD034
dicot8	IE	AtLBD027 EgraLBD025 VvinLBD007 VvinLBD038 PtriLBD057 PtriLBD027
dicot9	IA	AtLBD021 EgraLBD032 VvinLBD041 PtriLBD047 PtriLBD003
dicot10	IA	AtLBD010 AtLBD028 AtLBD036 PtriLBD037 PtriLBD024
dicot11	IC1/D	AtLBD003 EgraLBD016 VvinLBD024 PtriLBD039 PtriLBD022
dicot12	IC1/D	EgraLBD015 VvinLBD010 PtriLBD055 PtriLBD026
dicot13	IB	EgraLBD028 VvinLBD034 PtriLBD030 PtriLBD038
dicot14	IB	AtLBD020 EgraLBD026 VvinLBD035 PtriLBD013
dicot15	IB	AtLBD033 EgraLBD033 VvinLBD022 PtriLBD005
dicot16	IE	AtLBD022 PtriLBD050 PtriLBD017 PtriLBD019

Group	Class	LBD gene
dicot17	IB	AtLBD014 EgraLBD019 VvinLBD033 PtriLBD029
dicot18	IA	AtLBD006 PtriLBD048 PtriLBD004
dicot19	IC1/D	VvinLBD013 PtriLBD049 PtriLBD018
dicot20	IC1/D	EgraLBD009 PtriLBD002
dicot21	II	EgraLBD035 VvinLBD042
monocot1	IA	ZmayLBD036 ZmayLBD019 ZmayLBD033 ZmayLBD040 OsLBD011 OsLBD031 SbicLBD020 SbicLBD009
monocot2	IB	ZmayLBD007 ZmayLBD003 ZmayLBD042 OsLBD023 OsLBD001 SbicLBD032 SbicLBD031
monocot3	IB	ZmayLBD043 ZmayLBD001 ZmayLBD002 OsLBD024 OsLBD021 SbicLBD030 SbicLBD036
monocot4	IC1/D	ZmayLBD039 ZmayLBD004 ZmayLBD041 ZmayLBD029 OsLBD020 SbicLBD029 SbicLBD008
monocot5	IE	ZmayLBD009 ZmayLBD023 OsLBD004 OsLBD029 SbicLBD014 SbicLBD013
monocot6	II	ZmayLBD006 ZmayLBD014 OsLBD019 OsLBD005 SbicLBD027 SbicLBD016
monocot7	IB	ZmayLBD024 OsLBD028 OsLBD002 SbicLBD010 SbicLBD011
monocot8	IC1/D	ZmayLBD026 ZmayLBD012 OsLBD026 SbicLBD028
monocot9	IA	ZmayLBD010 ZmayLBD027 OsLBD027 SbicLBD033
monocot10	IC2	ZmayLBD020 OsLBD012 SbicLBD018
monocot11	IC1/D	ZmayLBD013 OsLBD025 SbicLBD035
monocot12	IC1/D	ZmayLBD032 OsLBD033 SbicLBD007
monocot13	II	ZmayLBD011 OsLBD022 SbicLBD026
monocot14	IB	ZmayLBD021 OsLBD014 SbicLBD021
monocot15	IC2	ZmayLBD044 OsLBD032 SbicLBD004

Group	Class	LBD gene
monocot16	IC1/D	ZmayLBD025 SbicLBD023 SbicLBD025
monocot17	II	ZmayLBD017 OsLBD018 SbicLBD022
monocot18	IA	ZmayLBD016 OsLBD013 SbicLBD019
monocot19	II	ZmayLBD037 ZmayLBD034 SbicLBD006
monocot20	IE	ZmayLBD018 SbicLBD024
monocot21	II	ZmayLBD005 SbicLBD034
monocot22	IC1/D	ZmayLBD008 SbicLBD012
monocot23	IE	ZmayLBD030 SbicLBD001
