# Supplementary figures and images for: Decipher the ancestry of the plant-specific LBD gene family
Source: BMC Genomics. 2017 Jan 25;18(Suppl 1):951. doi: 10.1186/s12864-016-3264-3 (PMC5310275; doi:10.1186/s12864-016-3264-3)

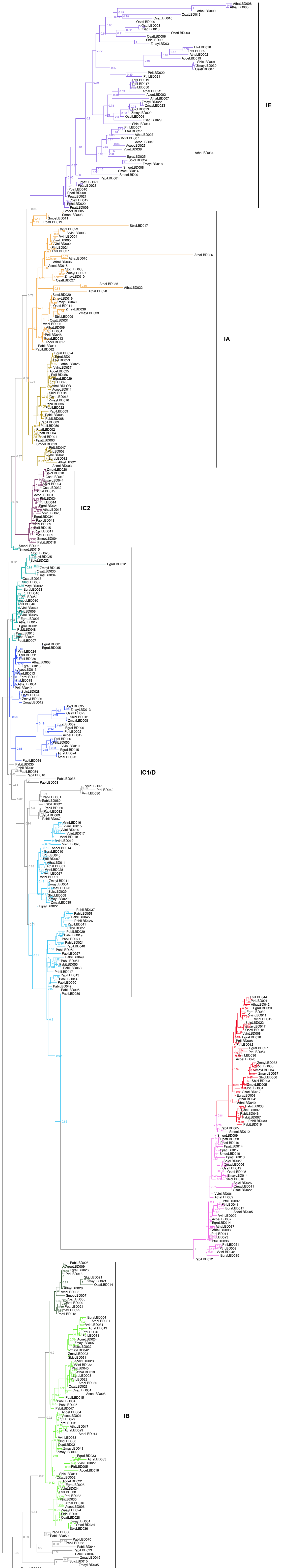

IE

IA

IC2

IC1/D

II

IB

Supplement: Additional file 3: — ML tree of LBD genes in land plants. (PDF 74 kb) [file 12864_2016_3264_MOESM3_ESM.pdf]

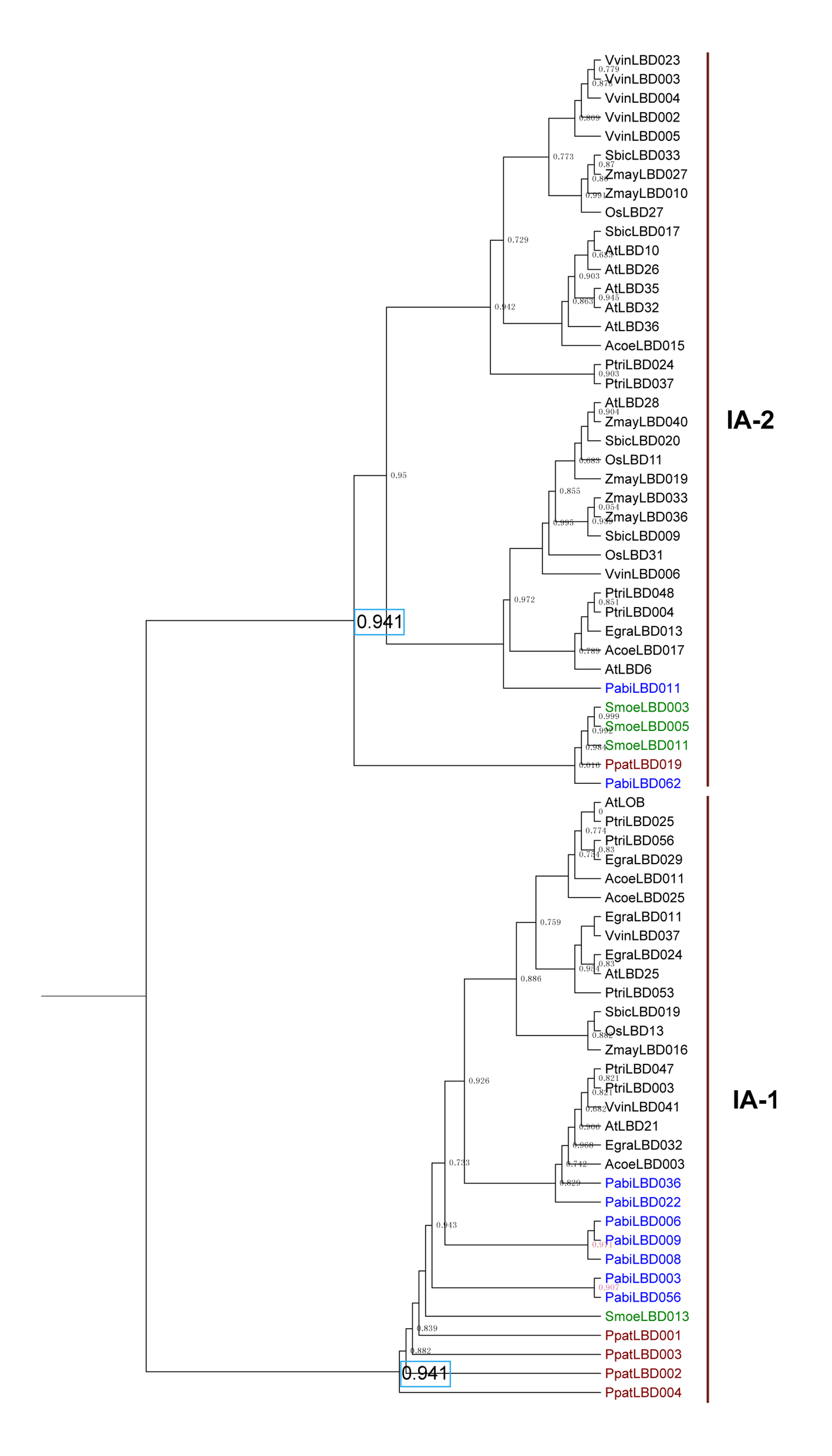

Supplement: Additional file 4: — ML tree of class IA LBD genes. (TIF 1275 kb) [file 12864_2016_3264_MOESM4_ESM.tif]

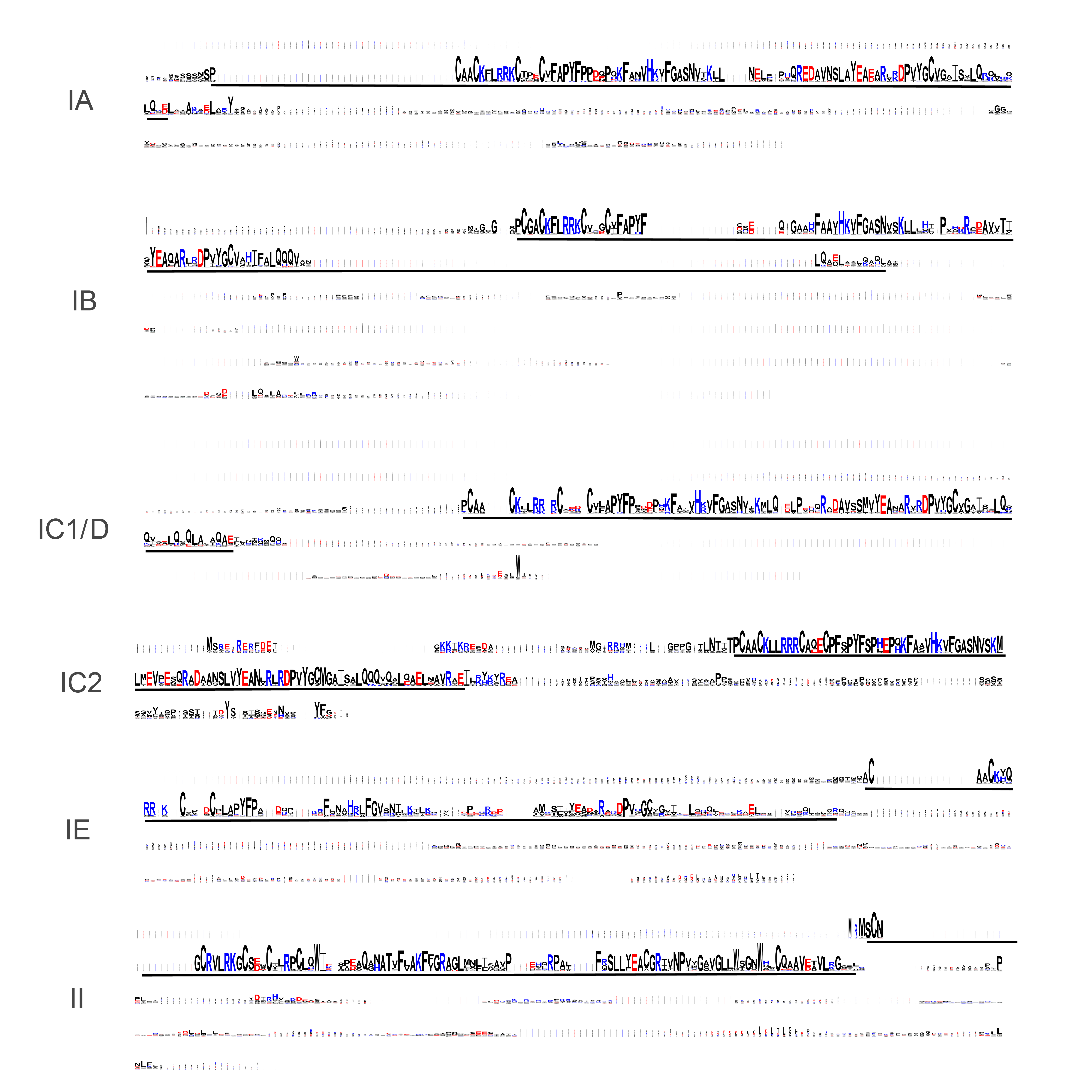

Supplement: Additional file 5: — Sequence logo of LBD proteins in each class. (TIF 2487 kb) [file 12864_2016_3264_MOESM5_ESM.tif]

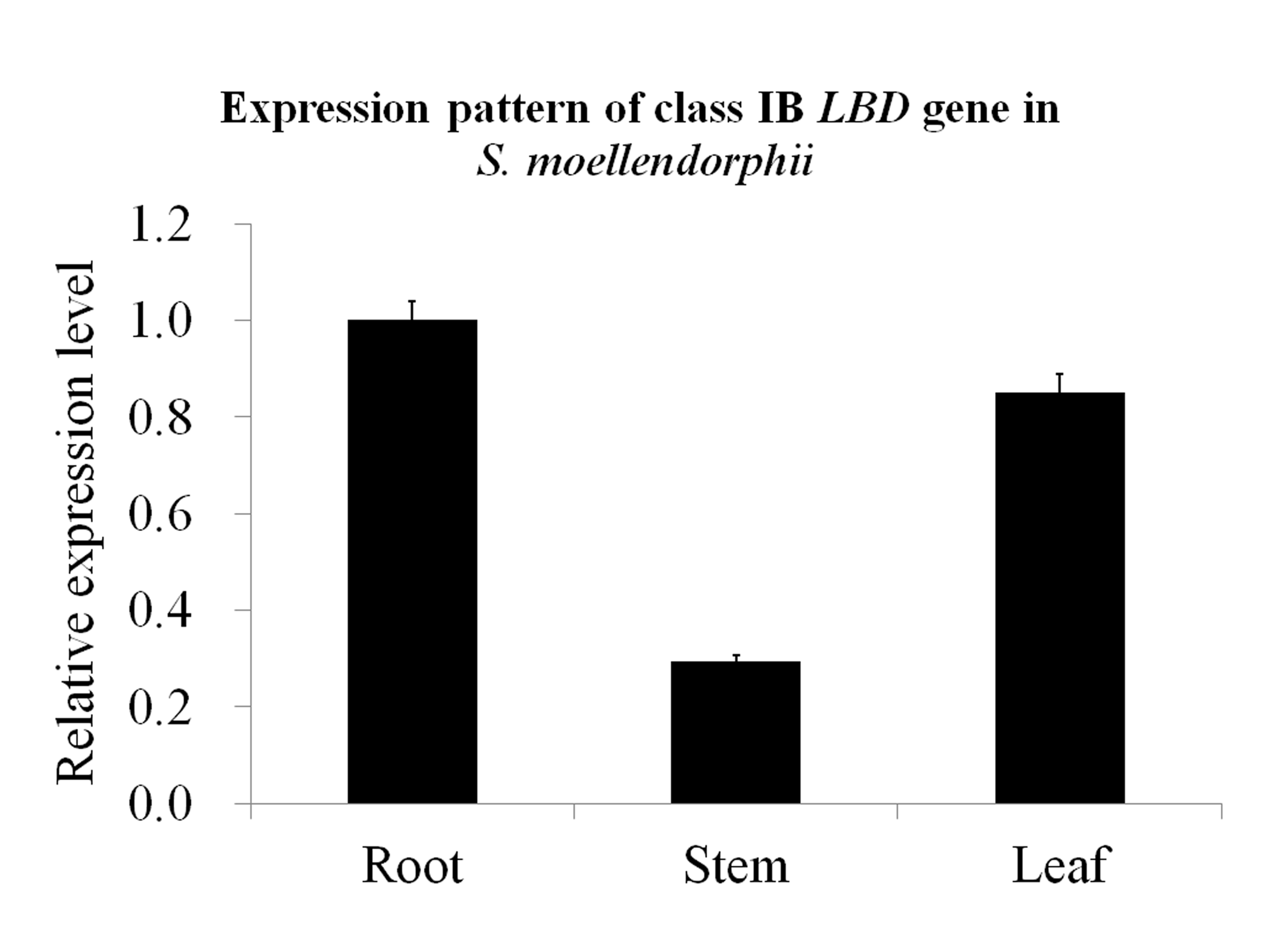

Supplement: Additional file 6: — Expression of class IB LBD gene in S. moellendorphii. (TIF 381 kb) [file 12864_2016_3264_MOESM6_ESM.tif]

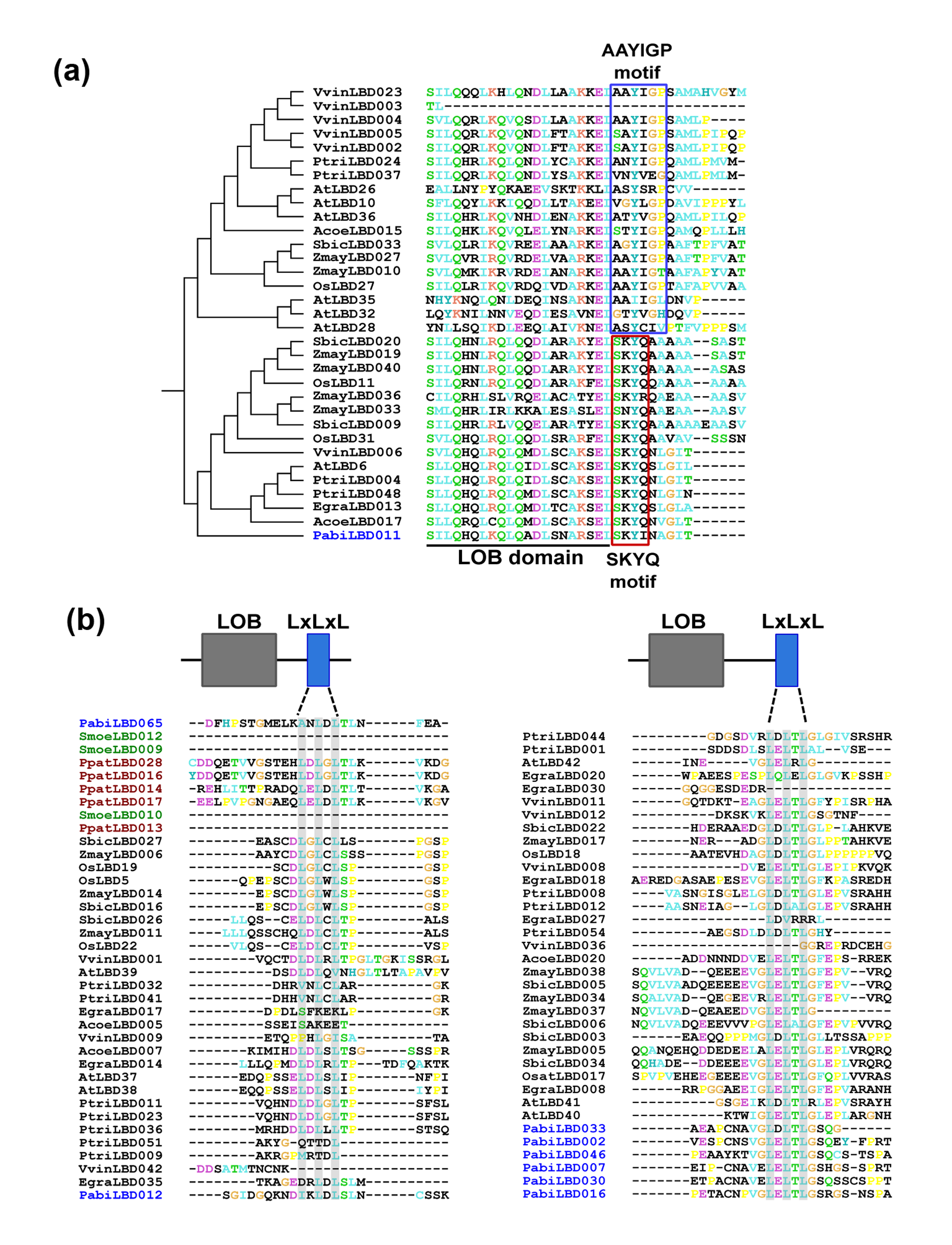

Supplement: Additional file 7: — Representative motifs for class IA and class II genes. (TIF 4927 kb) [file 12864_2016_3264_MOESM7_ESM.tif]
